# Neural information processing with dynamical synapses

**DOI:** 10.3389/fncom.2013.00188

**Published:** 2013-12-26

**Authors:** Si Wu, K. Y. Michael Wong, Misha Tsodyks

**Affiliations:** ^1^State Key Laboratory of Cognitive Neuroscience & Learning and IDG/McGovern Institute for Brain Research, Beijing Normal UniversityBeijing, China; ^2^Center for Innovation and Collaboration in Brain and Learning Sciences, Beijing Normal UniversityBeijing, China; ^3^Department of Physics, Hong Kong University of Science & TechnologyHong Kong, China; ^4^Department of Neurobiology, Weizmann Institute of ScienceRehovot, Israel

**Keywords:** short-term plasticity, phenomenological model, neural information processing, associative memory, network dynamics, neural field model, continuous attractor neural network

Experimental data have consistently revealed that the neuronal connection weight, which models the efficacy of firing of a pre-synaptic neuron in modulating the state of the post-synaptic neuron, varies on short time scales, ranging from tens to thousands of milliseconds (Markram and Tsodyks, 1996; Zucker and Regehr, 2002). This is called short-term plasticity (STP). Two types of STP, with opposite effects on the connection efficacy, have been observed in experiments, which are known as short-term depression (STD) and short-term facilitation (STF).

Computational studies have explored the impact of STP on single neuron and network dynamics, and found that STP can generate very rich intrinsic dynamical behaviors, including adaptation, temporal filtering, damped oscillation, state hopping with transient population spike, traveling front and pulse, spiral wave, rotating bump state, robust self-organized critical activity and so on. These studies also strongly suggest that STP may play many important roles in neural computation. For instances, STD may generate a dynamic control mechanism that allows equal fractional changes on rapidly and slowly firing afferents to produce post-synaptic responses, realizing Weber's law (Abbott et al., 1997); STD may generate a mechanism to close down network activity naturally, achieving iconic sensory memory (Fung et al., 2012); STD may provide a mechanism for memory searching by destabilizing attractor states (Torres et al., 2007); and STF may provide a mechanism for implementing work memory without recruiting neural firing (Mongillo et al., 2008).

From the computational point of view, the time scale of STP resides between fast neural signaling (on the order of milliseconds) and slow experience-induced learning (on the order of minutes or above), and it is on the time order of many important temporal processes occurring in our daily lives, such as motion control, speech recognition and working memory. Thus, STP may serve as a substrate for neural systems manipulating temporal information on the relevant time scales.

This *Research Topic* presents new results in the study of STP and summarizes some recent progress in the field. It includes the works on analyzing the phenomenological models of STP, the effects of STP on single neuron and network dynamics, and the roles of STP in a number of neural information processes.
